# Incidence, outcome, and attributable resource use associated with pulmonary and cardiac complications after major small and large bowel procedures

**DOI:** 10.1186/2047-0525-3-7

**Published:** 2014-10-07

**Authors:** Lee A Fleisher, Walter T Linde-Zwirble

**Affiliations:** 1 Department of Anesthesiology and Critical Care, Perelman School of Medicine, Leonard Davis Institute of Health Economics, University of Pennsylvania, 3400 Spruce Street, Dulles 680, Philadelphia, PA 19104, USA; 2 ZD Associates, Perkasie, PA, USA

**Keywords:** Colectomy, Surgery, Postoperative, Pulmonary complications, Myocardial infarction, Cardiac arrest, Pneumonia, Cost

## Abstract

**Background:**

Complications increase the costs of care of surgical patients. We studied the Premier database to determine the incidence and direct medical costs related to pulmonary complications and compared it to cardiac complications in the same cohort.

**Methods:**

We identified 45,969 discharges in patients undergoing major bowel procedures. Postoperative pulmonary and cardiac complications were identified through the use of International Classification of Diseases, Ninth Edition, Clinical Modification (ICD-9-CM) codes and through the use of daily resource use data. Pulmonary complications included pneumonia, tracheobronchitis, pleural effusion, pulmonary failure, and mechanical ventilation more than 48 h after surgery. Cardiac complications included ventricular fibrillation, acute myocardial infarction, cardiogenic shock, cardiopulmonary arrest, transient ischemia, premature ventricular contraction, and acute congestive heart failure.

**Results:**

Postoperative pulmonary complications (PPC) or postoperative cardiac complications (PCC) were present in 22% of cases; PPC alone was most common (19.0%), followed by PPC and PCC (1.8%) and PCC alone (1.2%). The incremental cost of PPC is large ($25,498). In comparison, PCC alone only added $7,307 to the total cost.

**Conclusions:**

The current study demonstrates that postoperative pulmonary complications represent a significant source of morbidity and incremental cost after major small intestinal and colon surgery and have greater incidence and costs than cardiac complications alone. Therefore, strategies to reduce the incidence of these complications should be targeted as means of improving health and bending the cost curve in health care.

## Background

During the past decade, there has been a marked decrease in surgical mortality [1]. This decrease appears to be related in part to better treatment of complications in some hospitals since perioperative complications remain high and continue to be a significant source of both decreased health and increased costs after surgery [2]. Beginning in the 1970s, there was a concerted effort to identify patients at increased risk of perioperative cardiovascular morbidity and mortality and to identify strategies to reduce these complications. This has led to the development and publication of formal Guidelines on Perioperative Cardiovascular Evaluation and Management for Noncardiac Surgery and incorporation of specific therapies into public performance measurement [3]. In contrast to cardiovascular disease, the evaluation and management of perioperative pulmonary complications has not had the same degree of concerted effort to either identify those at risk or implement additional risk reduction strategies.

The absence of a concerted focus on perioperative pulmonary complications may in part be due to the lack of recognition of their incidence and their economic burden [4]. An additional issue is related to the definition of pulmonary complications since there is wide variability in manifestations of complications: from minor aspiration to pneumonia to overt sepsis. Assessing the impact of pulmonary complications is also difficult since a pulmonary complication may lead to cardiovascular collapse with resultant cardiac morbidity or mortality. Pulmonary complications may also be the final common pathway for major morbidity or mortality in patients with other complications such as non-pulmonary infectious complications. Given the lack of concerted attention in this important area of health-care outcomes, we analyzed an enriched administrative dataset to determine the incidence and direct medical costs related to pulmonary complications in order to provide more focused attention on the problem and provide an impetus to identify cost-effective risk-reducing strategies and compared it to cardiac complications in the same cohort.

## Methods

### Case selection

We constructed the study cohort using the 2008 Premier Hospital Discharge database. The Premier database (Premier, Inc., Charlotte, NC, USA) includes information on all inpatients and hospital-based outpatients treated in more than 600 US hospitals. The database includes patient demographic data and diagnosis codes; the date-stamped log of all invoiced items, including procedures, medications, laboratory orders, diagnostic, and therapeutic services; as well as devices used by individual patients. The cohort of interest consisted of all discharges, age 18 years and older, in the Medicare Severity DRGs 329–331, major small and large bowel procedures. For each case, we extracted the following: demographic characteristics; principal diagnosis group (neoplasm, colitis/diverticulitis, other); Charlson-Deyo co-morbidities; postoperative pulmonary and cardiac complications; postoperative length of stay (LOS); postoperative intensive care unit (ICU) use; postoperative ICU LOS; total hospital costs; and national projection weights.

### Identification of postoperative complications

Postoperative pulmonary and cardiac complications were identified through the use of International Classification of Diseases, Ninth Edition, Clinical Modification (ICD-9-CM) codes in any secondary coding space and through the use of daily resource use data. Pulmonary complications included pneumonia (481, 482, 485, 486); tracheobronchitis (494.1, 466, 464.1); pleural effusion (511.1, 511.8, 511.9); pulmonary failure (518.81, 518.84); and mechanical ventilation more than 48 h after surgery (from daily resource use). Cardiac complications included ventricular fibrillation (427.4); acute myocardial infarction (410.X1); cardiogenic shock (785.51); cardiopulmonary arrest (427.5); transient ischemia (411.1, 411.89); premature ventricular contraction (PVC) (427.6); and acute congestive heart failure (428.21, 428.31, 428.41).

### Case-mix adjustment

We constructed a case-mix adjustment model to characterize the impact of preoperative characteristics on the incidence of postoperative complications and estimate the attributable effects of PPC and PCC on resource use and outcome. We constructed an analysis of variance model using patients with no PPC and no PCC to predict total cost as a function of age group; principal diagnosis group (neoplasm—150–59, 183, 188, 195, 197, 209, 211, 214, 228, 230–35, 239; colitis/diverticulitis—555–569; and other); and Charlson-Deyo co-morbidity. We chose cost and not mortality risk as a severity measure because hospital mortality is very low in those without postoperative complications. The predicted cost equation was applied to all cases, both those with and without postoperative complications, and subjects were characterized by quintiles of preoperative severity.

### Resource use and outcome presentation

Overall mortality and resource use was presented directly and also parsed into a base value, what was expected had there been no postoperative complications, and an incremental effect, the difference between the total and base values.

### Statistical analysis

We compared continuous data using the Mann–Whitney *U* test and categorical data by chi-squared or Fisher’s exact test as appropriate. We organized patient data by the presence of postoperative complications (PPC only, PCC only, neither PPC nor PCC, and both PPC and PCC) and by quintiles (Q) of preoperative severity. We generated national estimates using Premier-supplied weights. We constructed the databases in FoxPro (Microsoft Corp, Redmond, WA, USA) and conducted analyses in Data Desk (Data Description, Ithaca, NY, USA).

## Results

We identified 45,969 discharges with major small and large bowel procedures. The average age was 62.9 years and 45.1% were male. Descriptive characteristics are provided in Table 1. Colitis/diverticulitis was the most common principal diagnosis (43.7%), followed by neoplasm (39.3%) and other (17.0%). More than a third of the cohort (37.2%) had at least one Charlson-Deyo co-morbidity, with diabetes being the most common (15.8%) followed by metastatic neoplasm (13.1%) and chronic pulmonary conditions (8.0%).

**Table 1:** Major small and large bowel procedure cohort characteristic, overall and by postoperative complication group

**Variables**	**All**	**No PPC/PCC**	**PPC alone**	**PCC alone**	**PPC and PCC**
	**(*n*** **= 45,969)**	**(*n*** **= 35,875)**	**(*n*** **= 8,744)**	**(*n*** **= 547)**	**(*n*** **= 803)**
Rate (%)		78.0	19.0	1.2	1.8
Hospital mortality (%)	3.6	0.7	11.9	12.6	36.1
Age (mean, years)	62.9	61.3	67.8	73.7	74.5
Age group (%)					
18–34	5.4	6.2	3.2	0.9	0.6
35–54	23.8	26.5	15.6	6.2	5.7
55–64	21.3	22.3	18.5	14.3	10.8
65–74	22.2	21.7	23.7	23.9	25.7
75–84	19.7	17.6	26.0	35.8	37.0
85+	7.5	5.8	12.9	18.8	20.2
Sex, male (%)	45.1	45.0	45.3	49.2	46.8
Race (%)					
White	70.2	70.2	69.7	72.4	71.5
Black	10.4	10.0	12.0	11.2	12.0
Other	19.4	19.8	18.3	16.5	16.6
Charlson-Deyo co-morbidity (%)					
Diabetes	15.8	14.6	19.9	22.7	24.7
Complicated diabetes	1.4	1.1	2.3	2.2	3.1
Chronic pulmonary disease	8.0	5.9	16.0	12.6	16.6
Chronic renal disease	6.4	4.4	12.9	14.1	21.2
Neoplasm	5.7	5.0	8.5	5.3	7.2
Metastatic neoplasm	13.1	12.6	14.6	14.6	14.1
Cerebrovascular disease	1.1	0.9	1.9	1.5	2.7
Dementia	0.3	0.2	0.6	1.1	0.6
Prior myocardial infarction	4.0	3.5	5.0	11.5	10.5
Para- and quadriplegia	0.4	0.3	0.9	0.9	1.4
Peripheral vascular disease	3.1	2.3	5.5	9.9	9.2
Chronic rheumatic disease	2.2	1.9	3.2	2.5	3.2
Mild liver disease	0.9	0.7	1.7	1.1	1.7
Severe liver disease	0.4	0.2	1.1	0.4	1.2
HIV	0.1	0.1	0.1	0.0	0.0
Any co-morbidity	37.2	34.1	47.2	53.2	55.8
Mean number	0.6	0.5	0.9	1.0	1.2

### Incidence and survival

**Figure 1:**
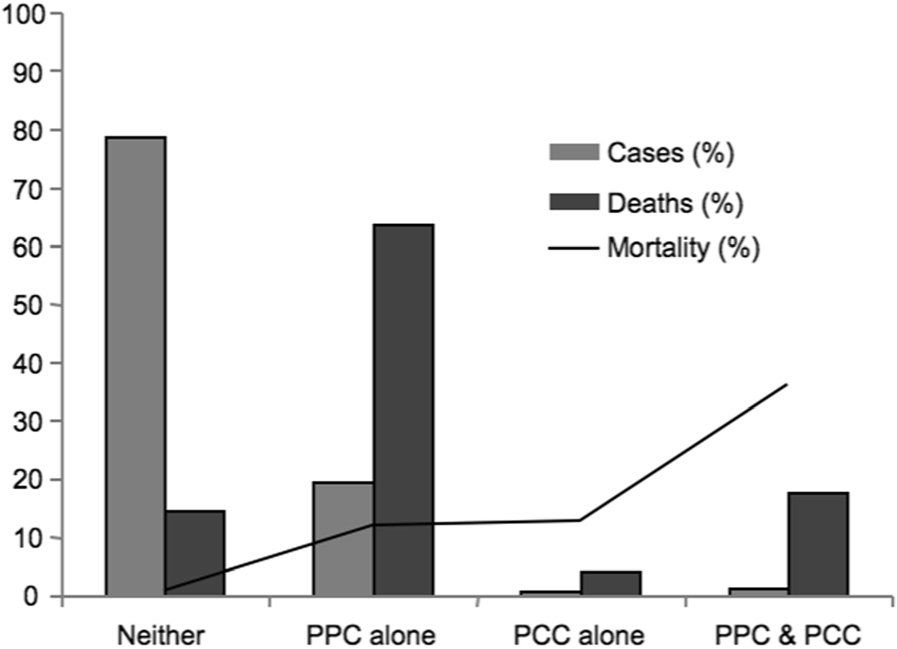
Incidence, case distribution, and mortality for postoperative complication groups.

Pulmonary or cardiac complications were present in 22% of cases; PPC alone was most common (19.0%), followed by PPC and PCC (1.8%) and PCC alone (1.2%). Those with postoperative complications were older and had a greater co-morbid burden than those that did not.Those with PPC alone had 11.9% mortality and accounted for nearly two thirds (63.6%) of hospital deaths (Figure 1). PCC alone cases had a similar mortality (12.6%) but accounted for only 4.2% of deaths. Those with PPC and PCC had the highest mortality (36.1%), though only 1.3% of the cohort accounted for 17.7% of deaths.

### Individual complications

Respiratory failure was the most common postoperative complication, being present in one in eight patients with 21.8% mortality (Table 2). Other pulmonary complications ranged from 0.4% incidence (pneumothorax) to 6.1% (MV48). Overall, 20.8% of patients had at least one PPC with a combined hospital mortality of 13.9%. Cardiac complications (2.9% incidence) were seven times less frequent than pulmonary complications, but with higher hospital mortality (26.6%). Acute myocardial infarction (AMI) was the most common PCC but occurred in only 1.2% of patients.

**Table 2:** Incidence and hospital mortality for postoperative complications

**Complications**	**Cases (% of group)**	**Rate (%)**	**Mortality (%)**
Pulmonary			
Pneumonia	2,249 (23.6)	4.9	14.4
Tracheobronchitis	434 (4.5)	0.9	6.2
Pleural effusion	1,584 (16.6)	3.4	13.7
Pulmonary collapse	3,499 (36.7)	7.6	6.2
Pneumothorax	189 (2.0)	2.0	24.9
Respiratory failure	5,686 (59.6)	12.4	21.8
MV48^a^	2,807 (29.4)	6.1	28.5
Any pulmonary	9,547	20.8	13.9
Cardiac			
Ventricular fibrillation	63 (4.7)	0.1	61.9
AMI	570 (42.2)	1.2	26.7
Cardiogenic shock	64 (4.7)	0.1	57.8
Cardiopulmonary arrest	220 (16.3)	0.5	70.9
Transient ischemia	47 (3.5)	0.1	2.1
PVC	367 (27.2)	0.8	6.0
Acute CHF	175 (13.0)	0.4	14.9
Any cardiac	1,350	2.9	26.6

^a^Mechanical ventilation more than 48 h after surgery.

### Severity model

The average cost of those without PPC and PCC increased with age ($2,732 more for those over 85 compared to those under 35), principal diagnosis of colitis/diverticulitis ($2,487 more than neoplasm), and co-morbidity (diabetes $1,091; peripheral vascular disease $1,493; cerebrovascular disease $4,792; chronic pulmonary disease $1,044; rheumatic disease $1,544; complex diabetes $2,700; para- and quadriplegia $3,422; chronic renal disease $6,046; and severe liver disease $8,706). The rate of postoperative complications increased greatly across the quintiles of severity (Table 3), increasing from 11.0% in the first quintile to 41.7% in the fifth quintile. While PPC alone tripled from 10.5% in Q1 to 34.7% in Q5, the rate of PCC alone and PPC and PCC increased by more than a factor of ten from Q1 to Q5.

**Table 3:** Association between preoperative severity, postoperative complications, and outcomes

**Severity quintiles**	**Q1**	**Q2**	**Q3**	**Q4**	**Q5**	**Total**
	**(*n*** **= 8,403)**	**(*n*** **= 9,976)**	**(*n*** **= 8,846)**	**(*n*** **= 9,174)**	**(*n*** **= 9,574)**	**(*n*** **= 45,969)**
Complication rate (%)						
PPC alone	10.5	14.5	13.9	20.3	34.7	19.0
PCC alone	0.2	1.3	0.8	1.3	2.2	1.2
PPC and PCC	0.3	1.1	1.0	1.4	4.7	1.7
Any complication	11.0	16.9	15.7	23.0	41.7	21.9
Base mortality (%)	0.1	0.4	0.3	0.7	2.2	0.7
Incremental mortality (%)						
PPC alone	5.2	7.9	8.3	8.5	15.8	11.2
PCC alone	3.1	7.5	6.4	8.7	15.6	12.0
PPC and PCC	13.5	35.0	33.8	32.9	36.3	35.5
Base cost ($)	14,875	15,438	16,017	17,462	20,784	16,672
Incremental cost ($)						
PPC alone	22,199	24,199	24,658	26,817	26,508	25,498
PCC alone	5,524	5,528	9,070	4,057	9,765	7,307
PPC and PCC	45,308	48,950	38,096	30,884	31,394	34,872
Base ICU use (%)	6.0	9.0	8.2	11.8	23.3	11.0
Incremental ICU use (%)						
PPC alone	42.0	45.7	50.2	54.0	52.7	54.0
PCC alone	9.8	24.9	33.1	20.4	25.2	28.5
PPC and PCC	71.2	67.1	75.3	68.3	59.9	70.6
Base post-op ICU LOS (days)	0.2	0.2	0.2	0.3	0.7	0.3
Incremental post-op ICU LOS (days)						
PPC alone	3.0	4.0	3.7	4.6	5.0	4.5
PCC alone	0.4	0.7	1.0	0.6	1.4	1.1
PPC and PCC	7.5	8.0	7.5	6.7	6.5	7.1
Base post-op floor LOS (days)	5.9	6.3	6.4	6.6	7.6	6.5
Incremental post-op floor LOS (days)						
PPC alone	3.0	4.0	3.7	4.6	5.0	4.5
PCC alone	0.4	0.7	1.0	0.6	1.4	1.1
PPC and PCC	7.5	8.0	7.5	6.7	6.5	7.1

### Hospital mortality and resource use by severity quintiles

**Figure 2:**
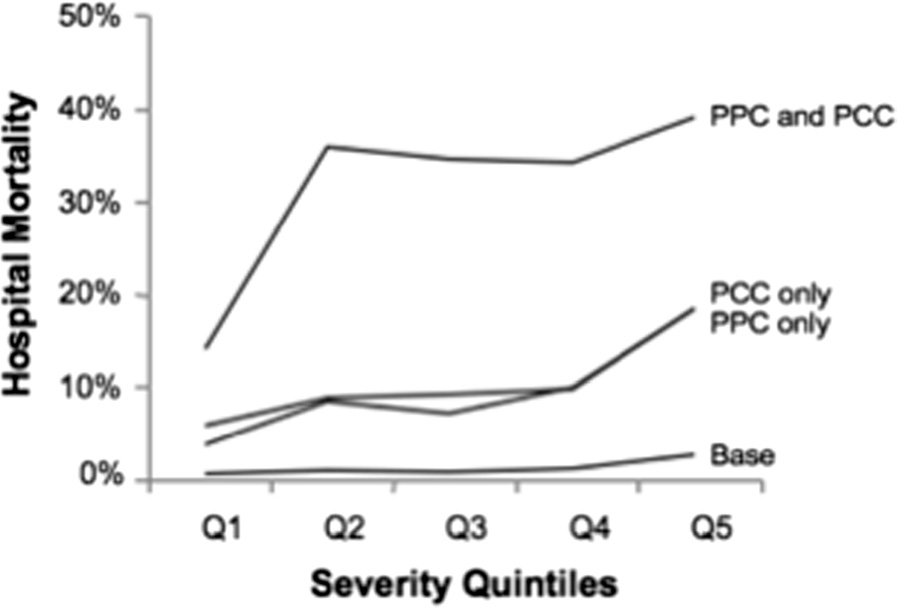
Base and incremental mortalities for postoperative complication groups by preoperative severity groups.

**Figure 3:**
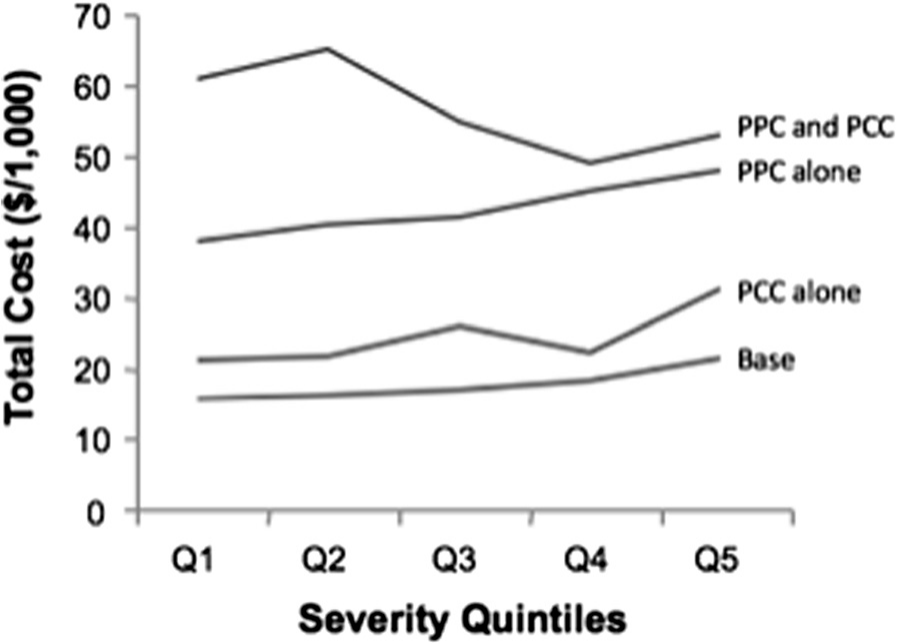
Base and incremental costs for postoperative complication groups by preoperative severity groups.

The base mortality increased from 0.1% in Q1 to 2.2% in Q5 for an overall value of 0.7% (Table 3). This is dwarfed by the incremental mortality associated with PPC alone (11.2% absolute), PCC alone (12.0%), and PPC and PCC (35.5%) (Figure 2). While the base cost increased by $5,909 between Q1 and Q5, the incremental cost of PPC is large ($25,498) but increased only modestly from $22,199 in Q1 to $26,508 in Q5 (Figure 3). In comparison, PCC alone only added $7,307 to the total cost. Overall, the incremental cost of PPC is 153% of the mean base cost.

In contrast to mortality and cost, the base ICU use rate increased greatly from 6.0% in Q1 to 23.3% in Q5. Those with postoperative complications had much greater ICU use. Postoperative ICU use for those with PPC rose from 48% in Q1 to 76% in Q5. This is much greater than PCC alone and less than PPC and PCC, which in Q5 had an ICU use rate of 83.2%. Base postoperative floor LOS ranged from 5.9 days in Q1 to 7.6 days in Q5 with a mean of 6.4 days. PPC alone added 3.5 days, greater than PCC alone (1.3 days) and PPC and PCC (2.8 days). Base postoperative ICU LOS was modest varying only from 0.2 days in Q1 to 0.7 in Q5. The incremental postoperative ICU LOS varied little by preoperative severity but could be large: adding 1.1 ICU days for PCC, 4.5 ICU days for PPC, and 7.1 ICU days for those with PPC and PCC.

### National projections

Projecting to US national levels, there were 308,798 major small and large bowel procedure discharges in 2008, with 59,980 with PPC alone, 3,739 with PCC alone, and 5,295 with PPC and PCC. The expected number of hospital deaths in the absence of PPC and PCC was 2,370, only 21% of the observed 11,157 deaths. The expected cost in the absence of postoperative complications was $5.25B, 75% of the observed $6.99B. Similarly, in the absence of PPC and PCC, we would have expected 51.5% of the observed ICU use, 90% of postoperative floor days, and 25% of ICU days to have been used. In summary, PCC alone was associated with 3.8% of deaths, PPC and PCC with 16.4% of deaths, and PPC alone with 58.5% of all deaths.

## Discussion

The current study demonstrates that postoperative pulmonary complications represent a significant source of morbidity and incremental cost after major small intestinal and colon surgery and have greater incidence and costs than cardiac complications alone. Although PPC alone was associated with a high incidence of mortality (11.9%), the combination of PPC and PCC together was associated with the greatest risk. The incidence increased with increasing age until 85 years of age, with over 20% of all patients experiencing some form of pulmonary complication.

The high incidence of pulmonary complications is consistent with studies utilizing the American College of Surgeons National Surgical Quality Improvement Program (ACS NSQIP), although the actual rate is higher than that in their selected population of hospitals [5]. This may reflect differences in the definition of PPC we used, which included a larger group of diagnoses, or it may reflect differences between hospitals enrolled in a quality improvement program compared to a more random sample. Importantly, we observed a 4.9% incidence of postoperative pneumonia and 6.1% incidence of mechanic ventilation greater than 24 h, which is consistent with other studies. For example, Kennedy and colleagues reported a 25.4% incidence of complications for surgery for colon cancer, with respiratory complications being the second most common complication after superficial site infection [6]. In contrast, Arozullah and colleagues observed a 1.5% incidence of postoperative pneumonia in a mixed group of major noncardiac surgery at 100 Veterans Administration Medical Centers [7]. The lower incidence of pneumonia in this population may reflect the type of surgery compared to a cohort of major abdominal surgery in our study.

We observed a high mortality associated with PPC, particularly those with concomitant PCC. Arozullah and colleagues observed a 21% incidence of 30-day mortality in those patients with pneumonia compared to our 14.4% incidence of in-hospital mortality. Jencks and colleagues demonstrated that patients with postoperative pneumonia have a high incidence of readmission, and therefore, our in-hospital mortality rate may not reflect the true 30-day incident [8]. Additionally, we observed a more contemporary cohort of non-Veterans Administration Medical Centers, and they may actually have a better failure-to-rescue rate.

We observed that postoperative cardiac complications continue to occur at low rates (2.9% overall incidence with 1.2% incidence of AMI) but are associated with a high mortality rate. This is consistent with other studies. For example, Dimick and colleagues at the University of Michigan found the incidence of 30-day cardiovascular complications to be 1% overall in a cohort of general and vascular surgery patients [9]. A 26.6% in-hospital mortality is consistent with most contemporary studies and suggests that while decreasing in frequency, cardiac complications still carry a significant mortality. When evaluating the mortality rate for either complication, it is important to isolate solitary complications from those that are combined.

PPC was associated with a significant increased length of stay and overall costs. Dimick and colleagues demonstrated a $62,704 average increment in costs in those patients with PPC in a mixed cohort of patients [9]. Khan and colleagues reported an increased hospital length of stay of 89% and increased costs by 55%, which is more consistent with our findings [10]. Thompson and colleagues studied abdominal surgical patients and demonstrated an increased hospital length of stay of 11 days and charges of $31,000 associated with PPC [11]. Short and colleagues utilized Medicare claims for the years 2005 through 2009 in six cancer resections and assessed the rate of complications using the Agency for Healthcare Research and Quality Patient Safety Indicators (PSI) [12]. They found that the rate of postoperative respiratory failure was 2.58% and increased costs by >50% for all cancer resections. Vaughan-Sarrazin and colleagues looked at cost of complications in general surgery patients in the Veterans Administration NSQIP as a means of building a business case for improving surgical quality [13]. The average cost for patients with no complication was $22,000, while the total costs for patients with a pulmonary embolism was $62,726 and increased to more than $115,000 for patients with failure to wean from the ventilator within 48 h of operation.

The significant medical and economic burden of postoperative pulmonary or cardiopulmonary complications clearly suggest that increased attention should be directed at reducing this complication. We included an analysis of cardiac complications in order to assess the importance of pulmonary complications in perspective. The past decade has seen numerous studies published which have evaluated interventions to reduce cardiac complications of noncardiac surgery [3]. Similar studies are lacking in the area of reducing the incidence and burden of pulmonary complications. Shander and colleagues have recently reported on a patient safety summit dedicated to risk-reducing and preventive strategies [4]. They have emphasized the paucity of data regarding such strategies as well as discussed potential technologies and strategies to reduce the incidence of ventilator-associated pneumonia.

Our study has several limitations related to the use of administrative claims to assess burden and costs of disease. By using the Premier database, we have the distinct advantage of daily resource utilization and an enriched set of diagnostic codes. However, we still have the inherent limitation of any such dataset with regard to the accuracy of the codes and inclusions of all potentially relevant variables. For example, we did not analyze smoking status given the inaccuracy of this variable in discharge data. With respect to outcome measures, hospitals may have different definitions of the individual outcomes and surveillance for such outcomes. We also made some assumptions regarding the sequence of cardiac versus respiratory complications that could influence our findings.

## Conclusions

We have demonstrated that postoperative pulmonary complications, either in isolation or in tandem with postoperative cardiac complications, occur at a high incidence after intestinal surgery and are associated with significant mortality and costs. Therefore, strategies to reduce the incidence of these complications should be targeted as means of improving health and bending the cost curve in health care.

## Competing interests

Dr. Linde-Zwirble received funding to purchase the Premier database and consulting fees to demonstrate the value of the database from Covidien, which manufactures products which could be used to reduce perioperative pulmonary complications. The Department of Anesthesiology and Critical Care at the University of Pennsylvania has received grants and equipment from Covidien, but Dr. Fleisher has not received any direct research funds or honorarium.

## Authors’ contributions

LAF conceived of the study, participated in its design and analysis, and drafted the manuscript. WTLZ participated in the design of the study, performed the statistical analysis, and helped to draft the manuscript. Both authors read and approved the final manuscript.
